# The effects of cyclic peptide [R4W4] in combination with first-line therapy on the survival of *Mycobacterium avium*

**DOI:** 10.3389/fcimb.2025.1547376

**Published:** 2025-04-16

**Authors:** Melissa Kelley, Kayvan Sasaninia, Ali Badaoui, Ira Glassman, Arbi Abnousian, Nadia Rai, Rakesh K. Tiwari, Vishwanath Venketaraman

**Affiliations:** ^1^ Department of Biomedical Sciences, College of Osteopathic Medicine of the Pacific, Western University of Health Sciences, Pomona, CA, United States; ^2^ Department of Biomedical and Pharmaceutical Sciences, Chapman University School of Pharmacy, Irvine, CA, United States; ^3^ Department of Pharmaceutics, Faculty of Pharmacy, The Islamia University of Bahawalpur, Bahawalpur, Pakistan; ^4^ Department of Biomedical Sciences, College of Osteopathic Medicine of the Pacific-Northwest, Western University of Health Sciences, Lebanon, OR, United States

**Keywords:** *Mycobacterium avium*, antimicrobial, cyclic peptide, antibiotics, macrophages

## Abstract

**Background:**

*Mycobacterium avium* (*M. avium*) is a nontuberculous mycobacterium (NTM) that can cause pulmonary and extrapulmonary infections mostly in immunocompromised individuals, such as those with HIV and diabetes. Traditionally, rifampicin (RIF) and azithromycin (AZ) have been used for a 12-month duration as first-line antibiotics against *M. avium*. Due to the increased multidrug resistance, novel ways, such as enhancement of macrophages response, are needed to provide adequate immune response required to clear *M. avium* infection.

**Methods and findings:**

In this study, we aim to study the effects of using THP-1 cells, which are monocyte-like cells, to induce a macrophage response and control *M. avium* infection when used in combination with traditional treatments such as RIF and AZ in free and liposomal forms. Traditional treatments’ effects are studied when used alone and in combination therapy with cyclic peptide [R4W4] (liposomal encapsulated and liposomal combination). Colony-forming units (CFU) counts were assessed for all samples 3 hours, 4 days, and 8 days post-treatment. A significant reduction in the intracellular viability of *M. avium* was observed when THP-1 cells were treated with liposomal combination [R4W4]+RIF and liposomal combination [R4W4]+AZ compared to when treated with liposomal RIF or liposomal AZ alone, respectively.

**Conclusion:**

Our findings show that liposomal combination [R4W4] is a promising adjuvant therapy to increase *M. avium* susceptibility to known antibiotics.

## 1 Introduction

The global prevalence of nontuberculous mycobacteria (NTM) infection is steadily rising (Adjemian et al., 2012; Wang et al., 2023; Wang et al., 2023). A retrospective review by Wang et al. found that in Southwest China, prevalence of NTM increased significantly each year between 2017-2022 (Wang et al., 2023). The most predominant species identified was *Mycobacterium avium*, followed by *Mycobacterium abscessus* and *Mycobacterium intracellulare* (Wang et al., 2023). *Mycobacterium avium* complex (MAC) is the most common group of NTM and *M. avium* subsp. a*vium* is the most clinically significant species within MAC (Busatto et al., 2019). *M. avium* are acid-fast slow-growing mycobacteria that inhabit a wide range of environmental sources including soil, animal reservoirs, and animal byproducts such as milk and food products (Busatto et al., 2019). As an opportunistic human pathogen, *M. avium* disproportionately impacts immunocompromised individuals like those with HIV or diabetes, as well as individuals with pre-existing lung disease like cystic fibrosis, chronic obstructive pulmonary disease (COPD), or prior *Mycobacterium tuberculosis* infection and cavitary lesions (Diel et al., 2018b; Cano Rodríguez et al., 2023; Wang et al., 2023).

*M. avium* may be transmitted to humans through contact, inhalation, and ingestion (Busatto et al., 2019). Infection control is mediated through a combination of innate and adaptive immunity in the host. *M. avium* primarily targets mononuclear phagocytes (Li et al., 2010). Like *M. tuberculosis*, *M. avium* is consumed by macrophages, utilizing a range of pathogenic tools to survive within the phagosome-lysosome (Li et al., 2010). *M. avium* can inhibit phagosome-lysosome fusion and inhibit key enzymes that function to generate damaging reactive oxygen species (ROS) (Kaczmarkowska et al., 2022). In immunocompromised individuals, such as those with HIV, macrophages fail to receive their activating signal from natural killer (NK) cells and T-lymphocytes to contain *M. avium* growth (Kaczmarkowska et al., 2022).

*M. avium* produces three primary diseases in humans: pulmonary MAC (MAC-PD), disseminated MAC (D-MAC), and MAC lymphadenitis (MAC-L) (Kaczmarkowska et al., 2022). *M. avium* specifically results in approximately 80% of pulmonary NTM diseases (Busatto et al., 2019; To et al., 2020). A systematic review found that patients with MAC-PD have a five-year all-cause mortality rate of 27% (95% CI 21.3-37.8%) (Diel et al., 2018a; Fujishima et al., 2023). The guideline-directed therapy in place by the American Thoracic Society (ATS), European Respiratory Society (ERS), European Society of Clinical Microbiology and Infectious Diseases (ESCMID), and Infectious Disease Society of America (IDSA) suggests a once-daily regimen of azithromycin (AZ), rifampicin (RIF) or rifabutin, and ethambutol for fibrocavitary or severe nodular MAC-PD, thrice-weekly for non-severe nodular MAC-PD, and the addition of an aminoglycoside, amikacin, injection for severe fibrocavitary cases (Ito et al., 2022). This regimen is intended to be continued for more than 12 months after sputum identification. The current guideline has a history of low success rate (61.4%) plagued by adverse effects, duration of treatment, and macrolide resistance (Diel et al., 2018b). Long-term amikacin therapy has a success rate of 75% and is associated with nephrotoxicity in 6.3% of patients and ototoxicity in 25% of patients (Lee et al., 2023). A recent study found that the addition of rifampicin to the macrolide regimen did not add to the antimycobacterial effect, nor did it reduce the emergence of macrolide resistance, further demonstrating the need for novel antimycobacterial therapies (Schildkraut et al., 2023). Clofazimine, a phenazine molecule, has demonstrated a synergistic effect against *M. avium* when added to amikacin, however, it possesses frequent side effects and there is currently no current recommendation for standardized dosage or duration of treatment (Haworth et al., 2017; McGuffin et al., 2017; Daley et al., 2020; Lee et al., 2023).

One such approach for treating multidrug-resistant bacteria involves the use of antimicrobial peptides (AMPs). These are naturally occurring peptides, consisting of 8 or more amino acids, are an essential part of the innate immune system across eukaryotes, including humans, animals, and plants. AMPs have broad-spectrum antimicrobial activity against bacteria, fungi, viruses, and parasites. They typically disrupt the cell membranes of pathogens, leading to rapid cell death, which lowers the risk of resistance development - a common issue with traditional antibiotics. Additionally, AMPs are generally less harmful to the host, as they selectively target microbial cells while sparing human cells. Due to their unique mode of action and natural origin, AMPs are considered safer than traditional antibiotics (Rathinakumar et al., 2009; Lei et al., 2019). They have employed targeted delivery to the site of infection to reduce adverse effects. However, they face limitations such as poor oral absorption and rapid metabolism, resulting in a short duration of effectiveness, necessitating multiple dosing through the injectable route of administration (Joo, 2012). Liposomal formulations have been found effective in overcoming several of these issues, offering enhanced drug delivery and reduced toxicity (Ferreira et al., 2021). Liposomes are spherical vesicles composed of phospholipids that enclose an inner aqueous layer (Maqbool et al., 2019). The inclusion of cholesterol in their composition increases stability and protects them from degradation (Chibowski and Szcześ, 2016). Their non-immunogenic properties have garnered significant scientific attention (Mehta et al., 2020), leading to their use in treating infections and in clinical applications, such as the FDA-approved liposomal formulations for Amphotericin B (Ambisome^®^, Abelcet^®^, and Amphotec^®^) and Amikacin (Arikayce^®^ Kit) (Bulbake et al., 2017; He et al., 2022). [R4W4] is an amphiphilic cyclic peptide containing four arginine and four tryptophan amino acids, demonstrating broad-spectrum antimicrobial activity (Findlay et al., 2010). [R4W4] can form ionic bonds with the negative charge on the bacterial membrane and perturb it through hydrophobic interactions with membrane lipids (Joo, 2012). The efficacy of [R4W4] has been proven against various human pathogens, including methicillin-resistant *Staphylococcus aureus*, *Escherichia coli*, *Klebsiella pneumonia*, *Pseudomonas aeruginosa*, *Mycobacterium tuberculosis*, and *M. avium* in recent studies (Oh et al., 2014; Hernandez et al., 2020; Sajid et al., 2022; Kelley et al., 2023). More specifically, our laboratory has recently demonstrated that [R4W4] reduces *M. avium* survival when added alongside RIF or AZ more significantly than either antibiotic alone (Kelley et al., 2023). In this study, we aim to evaluate the optimal delivery method for [R4W4]. We developed liposomes using dioleoyl phosphatidylcholine (DOPC), cholesterol, and 1,2-distearoyl-sn-glycero-3-phosphoethanolamine-N-[carbonyl-amino(polyethylene glycol)-2000] (DSPE-PEG2000) for effective delivery of [R4W4], RIF, and AZ. Our objective was to determine whether [R4W4] is more effective when delivered as a liposomal encapsulated formulation, a liposomal combination formulation with RIF or AZ, or whether the therapy is more effective in its liposomal form compared to the free formulation.

## 2 Materials and methods

### 2.1 Liposomal preparation and characterization

Liposomes were prepared using the thin film hydration method as reported elsewhere, with some modifications (Zhang, 2017). Lipids and 1,2-distearoyl-sn-glycero-3-phosphoethanolamine-N-[carbonyl-amino(polyethylene glycol)-2000] were purchased from Avanti Polar Lipids, Alabama, USA. Cholesterol and other chemicals for liposomal preparation were purchased from Miliporesigma (USA). Briefly, lipids and the drug were dissolved at different compositions (**Table 1**) in 15 mL of chloroform in a round-bottom flask, and the organic solvent was evaporated using a rotary evaporator under 500 mbar pressure, 90 RPM, and at 45 °C, which is above the phase transition temperature of the lipids. A fine thin film formed in the round-bottom flask after 5 to 6 hours, which was then dried overnight. Phosphate-buffered saline (PBS) was added to the flask until the lipid film was fully dispersed in the aqueous medium. After the hydration process, the liposomal formulation was sonicated for 30 minutes. The formulation was then passed through an extruder five times to achieve uniformity. Finally, the liposomal vesicles were stored at 4 °C. The size, zeta potential, and polydispersity index (PDI) of the liposomes were performed using dynamic light scattering (DLS) with a Zetasizer Nano ZS (Malvern Instrument Ltd., UK). The liposomal dispersion was added to a glass cuvette and placed in the instrument (temperature 25°C, light scattering angle 90°). A capillary cuvette was used to measure the zeta potential. All measurements were performed in triplicate (Laouini et al., 2012).

**Table 1:** Composition of lipids and drugs used in formulation preparation.

Formulation	Drug	DOPCE (mg)	Cholesterol (mg)	DSPE-mPEG-2000 (mg)	PBS (mL)
[R4W4] Liposomes	400 (μg)	25	7	10	10
Rifampicin Liposomes	5 (mg)	25	7	10	10
Azithromycin Liposomes	2 (mg)	25	7	10	10
Peptide-Rifampicin Liposomes	400 (μg)/5 (mg)	25	7	10	10
Peptide-Azithromycin Liposomes	400(μg)/2 (mg)	25	7	10	10
Blank Liposome	No drug/peptide	25	7	10	10

### 2.2 Bacterial processing

All experiments utilized a laboratory strain of *M. avium* obtained from KWIKSTIK™, which was derived from ATCC 25291™. The *M. avium* culture was cultivated in 7H9 media from Hi Media (Santa Maria, CA, USA). The bacterial culture flasks and 7H9 media supplemented with albumin dextrose complex (ADC) were maintained in a 37°C incubator with 5% CO_2_. Before harvesting, the absorbance of the *M. avium* cell culture was measured at an optical density of 600 nm. *M. avium* was then harvested and processed in a static environment (without shaking) until it reached a logarithmic phase of growth. To disintegrate bacterial clumps and create a single-cell suspension, harvested *M. avium* was centrifuged and rinsed with 1X phosphate-buffered saline (PBS). The washed *M. avium* was then vortexed with 3 mm sterile glass beads for 3-minute intervals to break up bacterial clumps. The vortexed solution was filtered through a 5 µm filter to remove any remaining bacterial aggregates. The processed *M. avium* was serially diluted, plated on 7H11 agar, and incubated at 37°C to determine the bacterial count. Aliquots of the processed stock were placed in individual tubes, which were stored in a -80°C freezer until needed. All procedures were carried out under aseptic conditions within a Class II biochemical safety cabinet.

### 2.3 THP cell differentiation, infection, and antibiotic treatment

THP-1 cells from ATCC were cultured in RPMI-1640 medium obtained from Millipore Sigma-Aldrich and maintained in a 37°C incubator with 5% CO_2_. The cells were harvested for subsequent experiments. Before initiating the experiments, THP-1 cells were enumerated using a hemocytometer and trypan blue stain. A poly-L-lysine solution was applied to each well of a 96-well tissue culture plate for 1 h. The harvested THP-1 cells were treated with a 10 ng/mL solution of phorbol 12-myristate 13-acetate (PMA). The PMA-treated THP-1 cells (2x10^5 per well) were then added to each well in the 96-well tissue culture plate. The plate was placed in a 37°C incubator with 5% CO_2_ overnight to facilitate the differentiation of cells into macrophages before Day 0. After overnight incubation, each well was examined under a microscope to confirm the formation of a differentiated monolayer of cells. The supernatant was subsequently removed from each well. RPMI with 10% FBS,

infected with *M. avium*, was added to each well at a 1:1 concentration of *M. avium* to THP-1 cells (2x10^5 *M. avium*: 2x10^5 THP-1 cells). Following this addition, the 96-well plate was placed in a 37°C incubator with 5% CO_2_ for 1 h. After the incubation period, the supernatant was discarded, and unphagocytosed bacteria were removed by washing with a 1X PBS solution three times. Once unphagocytosed bacteria were removed, fresh RPMI with 10% FBS was added to each well. Various treatments were then administered to their corresponding wells, including liposomal [R4W4], liposomal AZ, liposomal RIF, liposomal encapsulated RIF+[R4W4], liposomal encapsulated AZ+[R4W4], free [R4W4], free RIF, free AZ, free AZ and [R4W4] together, and free RIF and [R4W4] together, each at different concentrations as specified in **Table 2**. Free peptides and antibiotics were dissolved in nanopure water and sterilized through a 0.22 µm filter prior to treatment. Water was used as a vehicle control for untreated cells.

**Table 2:** Various treatments and treatment combinations used and their corresponding concentrations.

Treatment	Concentrations of Drug or formulation used (µg/mL)
Liposomal Cyclic Peptide	2, 4, 8
Liposomal Azithromycin	1, 2, 4
Liposomal Rifampicin	4, 8, 16
Liposomal Rifampicin + Liposomal Cyclic peptide (LC)	4, 8, 16 + 2, 4, 8
Liposomal Azithromycin + Liposomal Cyclic peptide (LC)	1, 2, 4 + 2, 4, 8
Liposomal Azithromycin-Cyclic Peptide (LE)	1, 2, 4 + 2, 4, 8
Liposomal Rifampicin-Cyclic Peptide (LE)	4, 8, 16 + 2, 4, 8
Free Cyclic Peptide	2, 4, 8
Free Azithromycin	1, 2, 4
Free Rifampicin	4, 8, 16
Free Azithromycin + Cyclic Peptide	1, 2, 4 + 2, 4, 8
Free Rifampicin + Cyclic Peptide	4, 8, 16 + 2, 4, 8

Each treatment category was duplicated within the 96-well plate. After adding the treatments to their respective wells, the plate was incubated at 37°C with 5% CO_2_ for 3 hours. On Day 0, a specific number of wells were terminated for analysis. Sections of the 96-well plate were terminated on Day 0, Day 4, and Day 8, while treatments were administered on Day 0, Day 3, and Day 6. To terminate each well, the supernatant was discarded, and ice-cold nano-pure water was added. Slight friction was applied to release the cells, and the entire contents were removed. The samples were spread onto MiddleBrook 7H11 Agar Medium and placed in a 37°C incubator without CO_2_ for 11 days. After incubation, colonies of *M. avium* were counted.

### 2.4 Statistical analysis

Data was analyzed using GraphPad Prism software version 9.5.1. Treatment categories were analyzed using [ANOVA]. A p-value < 0.05 was considered statistically significant.

## 3 Results

### 3.1 Liposomal formulation and characterization

The liposomal formulations were characterized, and the results are presented in **Table 3**. The physicochemical properties of the formulations revealed mean particle sizes ranging from 126 to 232 nm. The blank formulation exhibited the smallest size at 126 nm, while the [R4W4]-loaded formulation showed an increased size of 156 nm. The addition of antibiotics further increased the particle size, with RIF-loaded liposomes measuring 181 nm and AZ-loaded liposomes measuring 210 nm. When both the [R4W4] and RIF were incorporated, the size reached 220 nm. Similarly, [R4W4] and AZ showed a size of 232 nm, which is the largest among all the prepared formulations. The polydispersity index (PDI) values for all formulations ranged from 0.125 to 0.255, indicating stable formulations. Additionally, the zeta potential of the formulations ranged from -3 to -7 mV, with these negative values suggesting reduced toxicity toward biological membranes. The cyclic peptide [R4W4] used in the formulations was synthesized using Fmoc/tBu solid-phase synthesis and characterized as previously reported (Riahifard et al., 2018). In this studies, Liposomal Combination (LC) refers to the use of two or more distinct liposomal formulations administered together to evaluate their comparative efficacy. In contrast, a Liposomal Encapsulated (LE) formulation refers to a single liposomal formulation in which either the cyclic peptide, a drug (AZ or RIF), or both are encapsulated within the liposomal structure. This study incorporates both approaches to assess their effectiveness in combating *M. avium* infections.

**Table 3:** Characterization results of liposomal formulation.

Formulations	Size (nm)	PDI	Zeta Potential (mV)
Peptide Liposomes	156.2 ± 0.5	0.255​ ± 0.02	-7​​ ± 0.57
Rifampicin Liposomes	181 ​± 5.2	0.151​​ ± 0.01	-6 ​± 1.15
Azithromycin​ Liposomes	210 ± 2.3	0.125 ​± 0.01	-3 ± 0.57
Peptide-Rifampicin Liposomes	220 ​± 8.27	0.214 ​​± 0.03	-4​ ± 1.15
Peptide-Azithromycin Liposomes	232 ± 1.36	0.162 ​​± 0.06	-5 ​± 1.23
Blank Formulation	126 ​± 5.24	0.142 ​​± 0.04	-7 ​​± 1.15

Each value represents the mean ± standard deviation of three measurements.

### 3.2 Survival of *M. avium* inside THP-1 macrophages treated with free form [R4W4]

**Figure 1:**
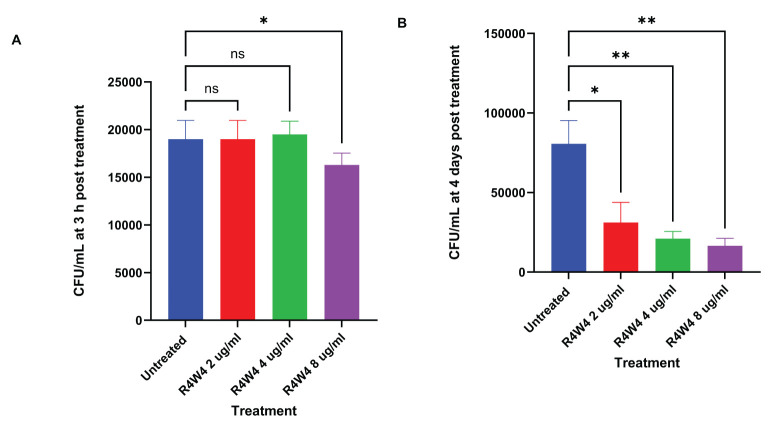
CFU counts of *M. avium* culture inside THP-1 cells treated with free form [R4W4]. **(A)** CFU/mL of *M. avium* treated with 2, 4, 8 µg/mL free [R4W4] at 3 h post-treatment. **(B)** CFU/mL of *M. avium* treated with 2, 4, 8 µg/mL free [R4W4] at 4 days post-treatment. *M. avium* was treated, then incubated at 37°C, and then terminated at 3 h and 4 days post-treatment. GraphPad Prism Software version 9.5.1 was utilized for analysis. Statistical analysis was performed using ANOVA. p-values are indicated at the top of each graph, and <0.05 (*) and <0.01 (**) were considered significant. Nonsignificant p-values are indicated as ns.

In this study, we examined the antimicrobial effects of [R4W4] alone against *M. avium* to demonstrate its baseline antimicrobial activity on *M. avium* survivability. We evaluated *M. avium* survivability with untreated, serving as our control, and increasing concentrations of [R4W4] at 3 h and 4 d post-treatment. At 3 h post-treatment, [R4W4] demonstrated no significant reduction in *M. avium* colonies at 2 µg/mL and 4 µg/mL compared to control. However, [R4W4] at 8 µg/mL exhibited a significant reduction in *M. avium* colonies 3 h post-treatment (**Figure 1A**; **Table 4**). By day 4 post-treatment, [R4W4] showed a significant reduction in *M. avium* colonies at all 3 concentrations (**Figure 1B**; **Table 4**).

**Table 4:** Mean CFU Counts of M. avium treated with 2, 4, 8 µg/mL free [R4W4] at 3 h post-treatment (A) and at 4 days post-treatment (B).

A		B	
Treatment (µg/mL)	Mean CFU	Treatment (µg/mL)	Mean CFU
Untreated	19000	Untreated	80700
R4W4 2	19000	R4W4 2	31200
R4W4 4	19500	R4W4 4	21100
R4W4 8	16300	R4W4 8	16600

### 3.3 Survival of *M. avium* inside THP-1 macrophages treated with free forms of rifampicin (RIF) and combination [R4W4]+RIF

**Figure 2:**
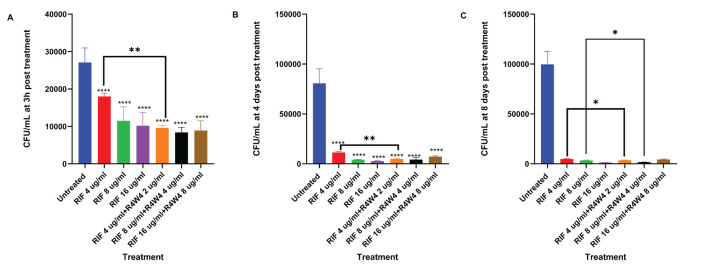
CFU counts *M. avium* culture inside THP-1 cells treated with free forms of RIF and combination [R4W4]+RIF. **(A)** CFU/mL of *M. avium* treated with 4, 8, 16 µg/mL free RIF and with 4, 8, 16 µg/mL free RIF with 2, 4, 8 µg/mL [R4W4], respectively, at 3 h post-treatment. **(B)** CFU/mL of *M. avium* treated with 4, 8, 16 µg/mL free RIF and with 4, 8, 16 µg/mL free RIF with 2, 4, 8 µg/mL [R4W4], respectively, at 4 days post-treatment. **(C)** CFU/mL of *M. avium* treated with 4, 8, 16 µg/mL free RIF and with 4, 8, 16 µg/mL free RIF with 2, 4, 8 µg/mL [R4W4], respectively, at 8 days post-treatment. GraphPad Prism Software version 9.5.1 was utilized for analysis. Statistical analysis was performed using ANOVA. p-values are indicated at the top of each graph, and <0.05 (*) and <0.01 (**) were considered significant. ****=P value <0.0001.

We assessed the antimicrobial activity of RIF with and without adjunctive [R4W4] to determine if the addition of [R4W4] led to a significant reduction in *M. avium* survivability when compared to RIF alone. We again employed our control as untreated and evaluated increasing concentrations of RIF, as well as increasing concentrations of combination [R4W4]+RIF in their free forms at 3 h, 4 d, and 8 d post-treatment. At both 3 h and 4 days post-treatment, all treatments exhibited a significant reduction in *M. avium* colonies when compared to the untreated sample. Furthermore, each treatment demonstrated a more pronounced reduction in *M. avium* colonies at 4 days post-treatment than at 3 h post-treatment. Combination [R4W4]+RIF exhibited a significant reduction in *M. avium* colonies at RIF 4 µg/mL + [R4W4] 2 µg/mL when compared to RIF 4 µg/mL alone at both 3 h and 4 days post-treatment. However, other combination treatment concentrations did not display a significant reduction in *M. avium* colonies when compared to their respective treatment of free RIF at 3 h and 4 days post-treatment (**Figures 2A, B**; **Table 5**).

**Table 5:** Mean CFU Counts of *M. avium* treated with 4, 8, 16 µg/mL free RIF and with 4, 8, 16 µg/mL free RIF with 2, 4, 8 µg/mL [R4W4], respectively, at 3 h post-treatment (A), 4 days post-treatment (B), and 8 days post-treatment (C).

A		B		C	
Treatment (µg/mL)	Mean CFU	Treatment (µg/mL)	Mean CFU	Treatment (µg/mL)	Mean CFU
Untreated	27100	Untreated	80700	Untreated	99700
RIF 4	18000	RIF 4	11400	RIF 4	4910
RIF 8	11500	RIF 8	4200	RIF 8	3360
RIF 16	10200	RIF 16	2700	RIF 16	1250
RIF 4 + R4W4 2	9600	RIF 4 + R4W4 2	4900	RIF 4 + R4W4 2	3600
RIF 8 + R4W4 4	8400	RIF 8 + R4W4 4	4300	RIF 8 + R4W4 4	1700
RIF 16 + R4W4 8	8900	RIF 16 + R4W4 8	7200	RIF 16 + R4W4 8	4400

By 8 days post-treatment, all treatments showed a significant reduction in *M. avium* colonies compared to the untreated sample. Combination [R4W4]+RIF continued to exhibit a significant reduction of *M. avium* colonies at RIF 4 µg/mL + [R4W4] 2 µg/mL compared to RIF 4 µg/mL alone. Similarly, combination [R4W4]+RIF demonstrated a significant reduction of *M. avium* colonies at RIF 8µg/mL + [R4W4] 4 µg/mL compared to RIF 8 µg/mL alone at 8 days post-treatment. However, RIF 16µg/mL + [R4W4] 8 µg/mL showed no statistical significance when compared to RIF 8 µg/mL alone at 8 days post-treatment (**Figure 2C**; **Table 5**).

### 3.4 Survival of *M. avium* inside THP-1 macrophages treated with liposomal forms of RIF, liposomal combination [R4W4]+RIF (LC), and liposomal encapsulated [R4W4]+RIF (LE)

**Figure 3:**
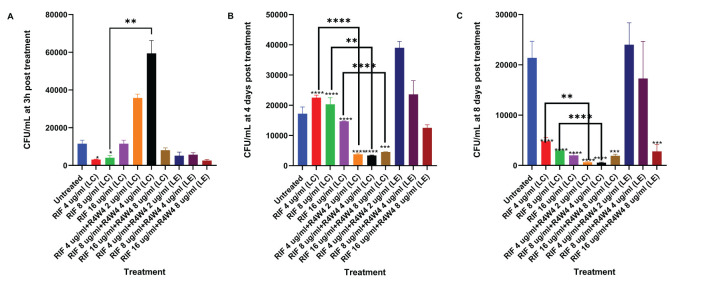
CFU counts of *M. avium* culture inside THP-1 cells treated with liposomal forms of RIF, liposomal combination [R4W4]+RIF (LC), and liposomal encapsulated [R4W4]+RIF (LE). **(A)** CFU/mL of *M. avium* treated with 4, 8, 16 µg/mL liposomal RIF and with 4, 8, 16 µg/mL liposomal RIF combination (LC) and encapsulated (LE) with 2, 4, 8 µg/mL [R4W4], respectively, at 3 h post-treatment. **(B)** CFU/mL of *M. avium* treated with 4, 8, 16 µg/mL liposomal RIF and with 4, 8, 16 µg/mL liposomal RIF combination (LC) and encapsulated (LE) with 2, 4, 8 µg/mL [R4W4], respectively, at 4 days post-treatment. **(A–C)** CFU/mL of *M. avium* treated with 4, 8, 16 µg/mL liposomal RIF and with 4, 8, 16 µg/mL liposomal RIF combination (LC) and encapsulated (LE) with 2, 4, 8 µg/mL [R4W4], respectively, at 8 days post-treatment. GraphPad Prism Software version 9.5.1 was utilized for analysis. Statistical analysis was performed using ANOVA. p-values are indicated at the top of each graph, and <0.05 (*), <0.01 (**), and <0.0001 (****) were considered significant.

In order to assess the optimal combination of RIF and [R4W4], we proceeded to compare the liposomal combination with the liposomal encapsulated formulation of RIF and [R4W4]. Utilizing the same concentration titration and time intervals, we separated the [R4W4]+RIF into liposomal combination and liposomal encapsulated formulations. At 3 h post-treatment, only liposomal RIF at concentrations of 4 µg/mL and 8 µg/mL exhibited a statistically significant reduction in *M. avium* colonies compared to the untreated sample. None of the combination treatments demonstrated a statistically significant reduction in *M. avium* colonies at 3h when compared to their corresponding concentration of RIF alone (**Figure 3A**; **Table 6**). However, liposomal combination [R4W4] 4 µg/mL + RIF 8 µg/mL (LC) showed a significant increase in *M. avium* colonies compared to RIF 8 µg/mL (LC) alone.

**Table 6:** Mean CFU Counts of *M. avium* treated with 4, 8, 16 µg/mL liposomal RIF and with 4, 8, 16 µg/mL liposomal RIF combination (LC) and encapsulated (LE) with 2, 4, 8 µg/mL [R4W4], respectively, at 3 h post-treatment (A), 4 days post-treatment (B), and 8 days post-treatment (C).

A		B		C	
Treatment (µg/mL)	Mean CFU	Treatment (µg/mL)	Mean CFU	Treatment (µg/mL)	Mean CFU
Untreated	11500	Untreated	17200	Untreated	21500
RIF 4 (LC)	3100	RIF 4 (LC)	22500	RIF 4 (LC)	4800
RIF 8 (LC)	4100	RIF 8 (LC)	20300	RIF 8 (LC)	3000
RIF 16 (LC)	11500	RIF 16 (LC)	14700	RIF 16 (LC)	2000
RIF 4 + R4W4 2 (LC)	35800	RIF 4 + R4W4 2 (LC)	3750	RIF 4 + R4W4 2 (LC)	640
RIF 8 + R4W4 4 (LC)	59400	RIF 8 + R4W4 4 (LC)	3350	RIF 8 + R4W4 4 (LC)	570
RIF 16 + R4W4 8 (LC)	8100	RIF 16 + R4W4 8 (LC)	4500	RIF 16 + R4W4 8 (LC)	1910
RIF 4 + R4W4 2 (LE)	5200	RIF 4 + R4W4 2 (LE)	39000	RIF 4 + R4W4 2 (LE)	24000
RIF 8 + R4W4 4 (LE)	5700	RIF 8 + R4W4 4 (LE)	23600	RIF 8 + R4W4 4 (LE)	17300
RIF 16 + R4W4 8 (LE)	2600	RIF 16 + R4W4 8 (LE)	12500	RIF 16 + R4W4 8 (LE)	2800

By 4 days post-treatment, all liposomal combination treatments of [R4W4]+RIF (LC) displayed a statistically significant reduction in *M. avium* colonies compared to their respective concentrations of liposomal RIF alone. However, liposomal encapsulated [R4W4]+RIF (LE) did not show any statistically significant reduction in *M. avium* colonies compared to their respective concentrations of RIF alone (**Figure 3B**; **Table 6**).

At 8 days post-treatment, liposomal combination treatments of [R4W4]+RIF (LC) at RIF 4 µg/mL + [R4W4] 2 µg/mL and at RIF 8 µg/mL + [R4W4] 4 µg/mL (LC) showed a statistically significant reduction in *M. avium* colonies compared to liposomal RIF 4 µg/mL and 8 µg/mL alone, respectively. However, liposomal combination treatments of [R4W4]+RIF (LC) at concentration of RIF 16 µg/mL + [R4W4] 8 µg/mL (LC) did not show a statistically significant reduction in *M. avium* colonies compared to liposomal RIF 8 µg/mL alone. All liposomal encapsulated [R4W4]+RIF (LE) formulations did not show any statistically significant reduction in *M. avium* colonies compared to their respective concentrations of RIF alone (**Figure 3C**; **Table 6**). Next we turned our attention to another first-line therapy for comparison, Azithromycin.

### 3.5 Survival of *M. avium* inside THP-1 macrophages treated with free forms of azithromycin (AZ) and combination [R4W4]+AZ

**Figure 4:**
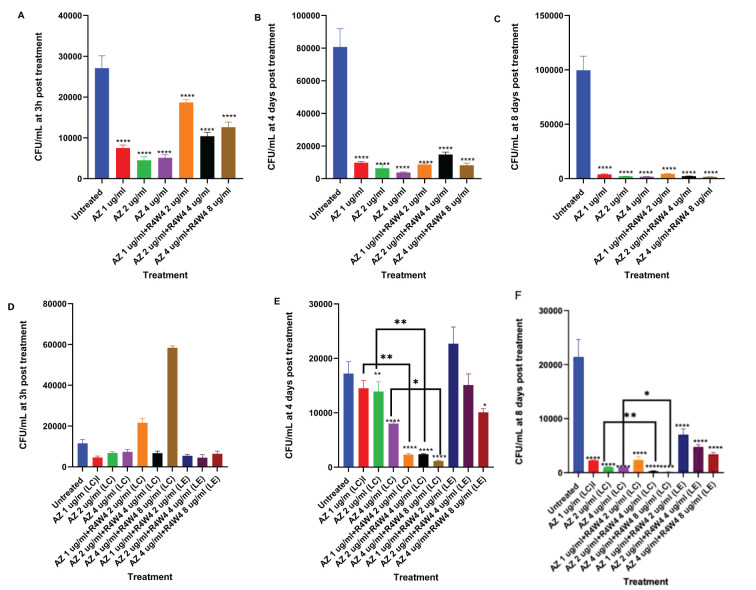
**(A–C)** CFU counts *M. avium* culture inside THP-1 cells treated with free forms of AZ and unconjugated [R4W4]+AZ. **(A)** CFU/mL of *M. avium* treated with 1, 2, 4 µg/mL free AZ and with 1, 2, 4 µg/mL free AZ with 2, 4, 8 µg/mL [R4W4] (unconjugated), respectively, at 3 h post-treatment. **(B)** CFU/mL of *M. avium* treated with 1, 2, 4 µg/mL free AZ and with 1, 2, 4 µg/mL free AZ with 2, 4, 8 µg/mL [R4W4] (unconjugated), respectively, at 4 days post-treatment. **(C)** CFU/mL of *M. avium* treated with 1, 2, 4 µg/mL free AZ and with 1, 2, 4 µg/mL free AZ with 2, 4, 8 µg/mL [R4W4] (unconjugated), respectively, at 8 days post-treatment. *M. avium* was treated, then incubated at 37°C and then terminated at 3 h, 4 days, and 8 days post-treatment. **(D–F)** CFU counts of *M. avium* culture inside THP-1 cells treated with liposomal forms of AZ, liposomal combination [R4W4]+AZ (LC), and liposomal encapsulated [R4W4]+AZ (LE). **(D)** CFU/mL of *M. avium* treated with 1, 2, 4 µg/mL liposomal AZ and with 1, 2, 4 µg/mL liposomal AZ combination (LC) and encapsulated (LE) with 2, 4, 8 µg/mL [R4W4], respectively, at 3 h post-treatment. **(E)** CFU/mL of *M. avium* treated with 1, 2, 4 µg/mL liposomal AZ and with 1, 2, 4 µg/mL liposomal AZ combination (LC) and encapsulated (LE) with 2, 4, 8 µg/mL [R4W4], respectively, at 4 days post-treatment. **(F)** CFU/mL of *M. avium* treated with 1, 2, 4 µg/mL liposomal AZ and with 1, 2, 4 µg/mL liposomal AZ combination (LC) and encapsulated (LE) with 2, 4, 8 µg/mL [R4W4], respectively, at 8 days post-treatment. GraphPad Prism Software version 9.5.1 was utilized for analysis. Statistical analysis was performed using ANOVA. p-values are indicated at the top of each graph, and <0.05 (*), <0.01 (**), and <0.0001 (****) were considered significant.

We began by evaluating the antimicrobial efficacy of AZ against *M. avium* survivability with and without [R4W4]. We evaluated M. avium survivability with untreated, serving as our control, and increasing concentrations of AZ and [R4W4]+AZ in their free forms at 3 h, 4 d, and 8 d post-treatment. At 3 h, 4 days, and 8 days post-treatment, there was no statistically significant decrease in *M. avium* cell counts observed for any of the treatment concentrations of AZ+[R4W4] compared to AZ alone at their respective concentrations (**Figures 4A–C**; **Table 7**). We then investigated whether AZ and [R4W4] demonstrated a significant difference in efficacy when in liposomal combination or liposomal encapsulated formulations.

**Table 7:** Mean CFU Counts of *M. avium* treated with 1, 2, 4 µg/mL free AZ and with 1, 2, 4 µg/mL free AZ with 2, 4, 8 µg/mL [R4W4] (unconjugated), respectively, at 3 h post-treatment (A), 4 days post-treatment (B), and 8 days post-treatment (C).

A		B		C	
Treatment (µg/mL)	Mean CFU	Treatment (µg/mL)	Mean CFU	Treatment (µg/mL)	Mean CFU
Untreated	27100	Untreated	80700	Untreated	99700
AZ 1	7500	AZ 1	9800	AZ 1	3930
AZ 2	4500	AZ 2	6400	AZ 2	2210
AZ 4	5100	AZ 4	3800	AZ 4	1860
AZ 1 + R4W4 2	18700	AZ 1 + R4W4 2	8700	AZ 1 + R4W4 2	4410
AZ 2 + R4W4 4	10400	AZ 2 + R4W4 4	14800	AZ 2 + R4W4 4	2290
AZ 4 + R4W4 8	12600	AZ 4 + R4W4 8	8200	AZ 4 + R4W4 8	1620

### 3.6 Survival of *M. avium* inside THP-1 macrophages treated with liposomal forms of azithromycin (AZ), liposomal combination [R4W4]+AZ (LC), and liposomal encapsulated [R4W4]+AZ (LE)

To determine the optimal delivery for AZ and [R4W4], we again tested the liposomal combination and liposomal encapsulated formulations to determine if there was a significant difference in *M. avium* survivability. Utilizing the same concentration titration and time intervals, we separated AZ and [R4W4]+AZ into liposomal combination and liposomal encapsulated formulations. At 3 h post-treatment, none of the AZ+[R4W4] combination treatments, both in encapsulated (LE) and combination (LC) formulations, demonstrated a statistically significant decrease in *M. avium* colonies compared to AZ alone at their respective concentrations (**Figure 4D**; **Table 8**).

**Table 8:** Mean CFU Counts of *M. avium* treated with 1, 2, 4 µg/mL liposomal AZ and with 1, 2, 4 µg/mL liposomal AZ combination (LC) and encapsulated (LE) with 2, 4, 8 µg/mL [R4W4], respectively, at 3 h post-treatment (A), 4 days post-treatment (B), and 8 days post-treatment (C).

A		B		C	
Treatment (µg/mL)	Mean CFU	Treatment (µg/mL)	Mean CFU	Treatment (µg/mL)	Mean CFU
Untreated	11500	Untreated	17200	Untreated	21400
AZ 1 (LC)	4600	AZ 1 (LC)	2250	AZ 1 (LC)	2250
AZ 2 (LC)	6800	AZ 2 (LC)	1030	AZ 2 (LC)	1030
AZ 4 (LC)	7300	AZ 4 (LC)	1030	AZ 4 (LC)	1030
AZ 1 + R4W4 2 (LC)	21600	AZ 1 + R4W4 2 (LC)	2360	AZ 1 + R4W4 2 (LC)	2360
AZ 2 + R4W4 4 (LC)	6700	AZ 2 + R4W4 4 (LC)	300	AZ 2 + R4W4 4 (LC)	300
AZ 4 + R4W4 8 (LC)	58400	AZ 4 + R4W4 8 (LC)	150	AZ 4 + R4W4 8 (LC)	150
AZ 1 + R4W4 2 (LE)	5400	AZ 1 + R4W4 2 (LE)	7040	AZ 1 + R4W4 2 (LE)	7040
AZ 2 + R4W4 4 (LE)	4500	AZ 2 + R4W4 4 (LE)	4760	AZ 2 + R4W4 4 (LE)	4760
AZ 4 + R4W4 8 (LE)	6400	AZ 4 + R4W4 8 (LE)	3400	AZ 4 + R4W4 8 (LE)	3400

By 4 days post-treatment, all liposomal combination treatments of [R4W4]+AZ (LC) exhibited a statistically significant reduction in *M. avium* colonies compared to liposomal AZ alone at their respective concentrations. However, all liposomal encapsulated treatments of [R4W4]+AZ (LE) did not display a statistically significant reduction in *M. avium* colonies compared to liposomal AZ alone at their respective concentrations (**Figure 4E**; **Table 8**).

At 8 days post-treatment, liposomal combination treatments of [R4W4]+AZ (LC) at AZ 1 µg/mL + [R4W4] 2 µg/mL (LC) did not show a significant reduction in *M. avium* colonies compared to liposomal AZ 1 µg/mL alone. Liposomal combination treatments of [R4W4]+AZ (LC) at AZ 2 µg/mL + [R4W4] 4 µg/mL (LC) and at AZ 4 µg/mL + [R4W4] 8 µg/mL (LC) demonstrated a statistically significant reduction in *M. avium* colonies compared to liposomal AZ 2 µg/mL and AZ 4 µg/mL alone, respectively. However, none of the liposomal encapsulated treatments of [R4W4]+AZ (LE) showed a statistically significant decrease in *M. avium* colonies compared to liposomal AZ alone at their respective concentrations (**Figure 4F**; **Table 8**).

## 4 Discussion

While guideline-directed therapy has led to a standardized treatment intended to contain infection, the increased use of first-line antibiotics yields drug-resistant strains of bacteria (Diel et al., 2018b; Ito et al., 2022). For example, RIF resistance in *M. tuberculosis* results from the mutated rifampicin resistance determining region (RRDR) of the *rpoB* gene (Rukasha et al., 2016; Muthaiah et al., 2017). The *rpoB* gene encodes the *M. tuberculosis* RNA polymerase (RNAP) beta subunit which is the target of RIF (Campbell et al., 2001; Muthaiah et al., 2017). AZ resistance arises most often from a point mutation at position 2058 or 2059 in the 23S ribosomal ribonucleic acid (rRNA) gene, which results in AZ being unable to bind ribosomes (Saxena et al., 2021; Nguyen and Daley, 2023). Recent research has explored the use of cyclic peptides as treatment options for multidrug-resistant bacterial infections. Several cyclic peptides have been studied against mycobacteria, including Ecumicin, Cyclomarin A, and Lassomycin (Gao et al., 2015; Jin et al., 2016; Maurer et al., 2019; Zhu et al., 2019). Our laboratory has previously demonstrated that [R4W4] is efficacious against *M. tuberculosis* when used in conjunction with first-line antibiotics isoniazid (INH) and pyrazinamide (PZA). We have also demonstrated that [R4W4] is efficacious against *M. avium* when used in conjunction with first-line antibiotics RIF and AZ (Hernandez et al., 2020; Kelley et al., 2023). There has yet to be research evaluating the optimal delivery method for adjunctive therapy of [R4W4].

This study aimed to determine the efficacy of [R4W4] in controlling the *M. avium* burden in THP-1 cells when used in combination with or encapsulated to first-line antibiotic therapy and when delivered in free form or a liposomal formulation. We used biocompatible lipids in the liposome composition, which presents a promising approach for drug delivery (Dymek and Sikora, 2022), particularly in pulmonary applications as reported by others (Elhissi, 2017). Liposomal formulations with DOPCE, Cholesterol, and DSPE-mPEG-2000 are particularly suited for drugs that are hydrophobic, prone to degradation, require controlled release, or have poor pharmacokinetic profiles. The inclusion of PEGylation helps in prolonging circulation time and reducing immunogenicity, making these formulations highly effective for targeted drug delivery and improving therapeutic outcomes (Dymek and Sikora, 2022). The small diameter of the prepared liposomes, especially those under 300 nm, offers a significant advantage in pulmonary drug delivery, as these smaller sizes have been shown to enhance drug deposition in the lungs (Chono et al., 2009; Mehta et al., 2020). The polydispersity index (PDI) values of all formulations were below 0.3, indicating the homogeneity of the prepared liposomes with minimal aggregation (Danaei et al., 2018). The zeta potential of the liposomes serves as an important indicator of formulation stability, with a negative charge contributing to reduced toxicity for biological membranes (Martins et al., 2013). These characteristics further support the potential of the prepared liposomal formulation for safe and effective pulmonary therapies. [R4W4] exerts its antibacterial effect due to its amphipathic nature and interaction with negatively charged phospholipids to perturb bacterial membranes (Oh et al., 2014). We first demonstrated the antibacterial effect of [R4W4] by treating THP-1 with free [R4W4] and observing CFU at 3 h and 4 days post-infection. These results are consistent with previous findings (Kelley et al., 2023).

In this study, we utilized THP-1-derived macrophages as an *in vitro* model to evaluate the intracellular efficacy of our liposomal cyclic peptide formulation in combination with first-line therapy against *M. avium*. While primary human alveolar macrophages would more closely mimic the *in vivo* lung environment, THP-1 cells are widely used in mycobacterial research due to their consistency, ease of culture, and ability to differentiate into macrophage-like cells that exhibit key functional properties, such as phagocytosis and intracellular bacterial survival. One limitation of using THP-1 cells is that they may not fully replicate the heterogeneity and immune responses of primary alveolar macrophages. However, the reproducibility and controlled conditions provided by THP-1 cells allow for reliable comparative analyses of treatment efficacy. Future studies utilizing primary human alveolar macrophages or *in vivo* models will be essential to further validate our findings and confirm the translational potential of this adjunct therapy for drug-resistant *M. avium*.

Our study revealed a significant reduction in the intracellular viability of *M. avium* when THP-1 cells were treated with liposomal formulations containing combination RIF+[R4W4] and AZ+[R4W4] when compared to liposomes containing either RIF or AZ alone, respectively. We found that in their free forms, there was no significant difference in *M. avium* survival when comparing AZ+[R4W4] versus AZ alone. For free-form RIF and RIF+[R4W4], there was a significant difference in *M. avium* survival when comparing 4 µg/mL RIF and 4 µg/mL RIF + 2 µg/mL [R4W4] at 3 hours, 4 days, and 8 days. There was also a significant difference between 8 µg/mL RIF and 8 µg/mL RIF + 4 µg/mL [R4W4] in only 8 days. These results are consistent with our previous findings demonstrating that cyclic peptide [R4W4] efficacy did not change at 8 µg/mL (Kelley et al., 2023).

When delivered in liposomal formulation, we observed a significant increase in synergy between [R4W4] and AZ or RIF as well as a significant difference in potency between liposomal encapsulated and liposomal combination forms. In **Figures 3A**, **4D**, the treatments with liposomal combinations of RIF or AZ and [R4W4] showed a 5-10 fold increase in CFU after 3 hours compared to pretreatment levels. Both experiments were conducted simultaneously, and this unexpected increase in CFU may be attributed to experimental errors, such as human error, or possibly to the excess lipids in the liposomal formulations. The presence of these lipids might have provided a growth substrate for the bacteria, particularly in cases where there was limited drug or peptide release from the liposomes. However, it is important to note that as the treatment progressed to 4 and 8 days, there was a marked reduction in CFU counts for these formulations compared to pretreatment levels. Thus, we believe it is valuable to include the original CFU data in the figures, even though it underscores an unusual observation in the early time points.

All concentrations of liposomal combination RIF+[R4W4] and AZ+[R4W4] demonstrated significant efficacy at 4 days when compared to either antibiotic alone. At 8 days, a significant reduction in *M. avium* survival was observed in liposomal combination 4 µg/mL RIF + 2 µg/mL [R4W4] and 8 µg/mL RIF + 4 µg/mL [R4W4] versus RIF alone, as well as liposomal combination 2 µg/mL AZ + 4 µg/mL [R4W4] and 4 µg/mL AZ + 8 µg/mL [R4W4] versus AZ alone. All liposomal encapsulated forms yielded no significant reduction in *M. avium* survival. The findings in our study demonstrate that potency of RIF and AZ against *M. avium* can be enhanced by delivering the antibiotics in a liposomal combination with [R4W4]. In contrast, the encapsulated liposomal formulation may exhibit altered release kinetics, likely due to the strong entrapment of antibiotics or [R4W4] within the liposome core, stemming from differences in their physicochemical properties. This could result in less effective synergistic interactions, leading to reduced therapeutic efficacy compared to the combination of separate liposomal formulations. The variability in the combinational effects of [R4W4] with RIF or AZ likely arises from several factors, including experimental conditions, the specific formulations used, and the pharmacokinetics of the drugs. Positive combinational effects were observed under conditions where the concentrations of RIF or AZ and the cyclic peptide, along with the release rates and uptake by macrophages, were optimal, resulting in enhanced bacterial killing. Conversely, antagonistic effects were observed in other conditions, which may be attributed to suboptimal drug release from the liposomal formulations or potential interference between the drugs and the peptide. For example, the liposomal formulations might have altered the bioavailability of the drugs, impacting their interaction with *M. avium*. Additionally, variations in macrophage uptake or the bacteria’s ability to utilize the liposomal lipids for growth could have further contributed to this inconsistency. Further studies are needed to optimize and validate the release of antibiotics and [R4W4] from the encapsulated liposomal preparation.

The findings of this study are presented with limitations. The strain of *M. avium* (*Mycobacterium avium* subsp. *avium*) employed in this study was obtained from the liver of a hen infected with *M. avium*, raising the possibility of constraints on the relevance of the findings to *M. avium* strains prevalent in humans (Sattar et al., 2021). Hence, we suggest conducting additional cyclic peptide [R4W4] studies specifically on *M. avium* subsp. *hominissuis*, the strain commonly identified in humans. This would help validate the effectiveness of cyclic peptide [R4W4] as a treatment approach for *M. avium* complex disease in human subjects (Shin and Shin, 2021).

Exploring novel approaches to test macrophage response opens avenues to monitor the effectiveness of emerging treatments in eliciting an appropriate macrophage response. In a study examining macrophage–pathogen interactions in zebrafish models, diverse methods were employed to assess macrophage functions. These methods included measuring reactive oxygen species (ROS) and reactive nitrogen species (RNS) response levels, calcium effluxes, apoptosis, and ATP usage (Torraca et al., 2014). Utilizing these techniques enables the evaluation of the efficacy of cyclic peptides in restraining *M. avium* infection by gauging the activation of macrophages.

Our findings suggest that the liposomal cyclic peptide formulation, when used in combination with RIF and AZ, has the potential to enhance treatment efficacy against *M. avium*, particularly in the context of drug-resistant infections. One of the major challenges in treating mycobacterial infections is the intracellular persistence of bacteria within macrophages, which limits drug penetration and reduces therapeutic efficacy. The use of liposomal encapsulation in our formulation improves drug delivery by enhancing cellular uptake, prolonging drug release, and maintaining therapeutic concentrations at the site of infection. Additionally, cyclic peptides have demonstrated antimicrobial activity through membrane disruption and potential inhibition of efflux pumps, mechanisms that could enhance the intracellular activity of rifampin and isoniazid. By increasing bacterial membrane permeability and reducing active drug efflux, the cyclic peptide component of our formulation may potentiate the effects of conventional antibiotics, thereby improving bacterial clearance. Furthermore, adjunctive combination therapy offers a strategy to combat antimicrobial resistance by targeting multiple bacterial pathways simultaneously. This reduces the selective pressure on individual drugs and minimizes the risk of resistance development. Given the increasing prevalence of drug-resistant *M. avium* and other nontuberculous mycobacterial infections, our approach represents a promising avenue for improving treatment outcomes.

In this study, we utilized *M. avium* of hen origin as a model to evaluate the efficacy of our liposomal cyclic peptide formulation in combination with first-line therapy. While there may be strain-specific differences between avian and human *M. avium* isolates, previous research has demonstrated that avian-derived *M. avium* strains share key genetic, phenotypic, and pathogenic characteristics with clinical human isolates. Notably, both avian and human strains exhibit similar mechanisms of intracellular survival, drug susceptibility patterns, and biofilm formation, making avian *M. avium* a suitable model for studying potential therapeutic interventions. However, we acknowledge that direct generalization to human infections requires further validation. Future studies utilizing clinical human *M. avium* isolates will be essential to confirm the translational relevance of our findings and further assess the efficacy of our liposomal formulation in a broader range of *M. avium* complex (MAC) infections. Despite this limitation, our results provide valuable insights into the potential application of liposomal cyclic peptides in combination therapy for drug-resistant *M. avium* infections.

Moving forward, the next steps for these findings involve assessing the effectiveness of cyclic peptide [R4W4] and combination treatments in the context of an active pulmonary *Mycobacterium avium* complex (MAC) infection using a murine model. Traditionally, C57BL/6, Balb/c, nude, and beige mice have been employed in *M. avium* infection studies (Verma et al., 2019). However, the C3HeB/Fej mouse strain has recently demonstrated necrotic foci during granuloma formation, resembling observations in humans and not typically seen in other mouse models. This makes the C3HeB/Fej mice a promising model for evaluating the efficacy of cyclic [R4W4] combination treatment during an active pulmonary MAC infection (Henao-Tamayo et al., 2015; Verma et al., 2019). [R4W4] could potentially be used both systemically and locally, depending on the delivery method. While systemic delivery would require further optimization to ensure safety and efficacy, localized delivery—such as targeting the lungs in MAC infections—could be a viable approach, particularly with liposomal formulations. To date, none of the preparations used in this study have been tested in animal models. Future work will focus on evaluating these formulations *in vivo* to determine their therapeutic potential and pharmacokinetics. Additionally, we aim to study the efficacy of our combination therapy when used on human-specific *M. avium* subspecies, such as *M. avium subsp. hominissuis* and *M. avium subsp. paratuberculosis* (Verma et al., 2019). Even though cytotoxicity doses have been documented *in vitro*, it is imperative to conduct randomized placebo-controlled clinical trials (RCTs) in healthy human subjects to comprehensively evaluate the safety and tolerability of the compound. Following the establishment of safety parameters, RCTs involving patients with active pulmonary *Mycobacterium avium complex* (MAC) infection are essential to substantiate cyclic [R4W4] as an adjunctive treatment for human MAC infection. This progression would pave the way for implementing these findings in the treatment of immunocompromised individuals with *M. avium* infection. However, careful consideration of safety, dosage volumes, and potential alterations in administration methods would be crucial in this transition.

## Data Availability

The original contributions presented in the study are included in the article/supplementary material. Further inquiries can be directed to the corresponding authors.
